# Natural microbiota confer life-long protection against obesity via an early-life, immune-mediated effect on brown adipocytes

**DOI:** 10.1080/19490976.2026.2718613

**Published:** 2026-08-23

**Authors:** Shahar Azar, Oksana Gavrilova, Greta Baker, Ji Hoon Oh, Keisuke Fukutomi, Jun Seishima, Jianping Ma, Moonhwa Kwak, Jonathan H. Badger, Giorgio Trinchieri, Benedikt Hild, Behdad Afzali, Margo P. Emont, Barbara Rehermann

**Affiliations:** a Immunology Section, Liver Diseases Branch, National Institute of Diabetes and Digestive and Kidney Diseases, National Institutes of Health, DHHS, Bethesda, MD, USA; b Mouse Metabolism Core, National Institute of Diabetes and Digestive and Kidney Diseases, National Institutes of Health, DHHS, Bethesda, MD, USA; c Immunoregulation Section, Kidney Diseases Branch, National Institute of Diabetes and Digestive and Kidney Diseases, National Institutes of Health, DHHS, Bethesda, MD, USA; d Laboratory of Integrative Cancer Immunology, Center for Cancer Research, National Cancer Institute, National Institutes of Health, DHHS, Bethesda, MD, USA; e Division of Biological Science, The University of Chicago, Chicago, IL, USA

**Keywords:** Natural microbiota, symbiont, commensal, wildling mice, early-life, metabolism, thermogenesis, energy expenditure

## Abstract

Obesity, a major risk factor for metabolic disease, has increased in industrialized nations alongside a reduction in gut microbiota diversity. Reconstituting laboratory mice with complex, natural microbiota and commensals from wild mice resulted in protection against diet-induced obesity. These natural microbiota reduced weight gain in both male and female mice of different genetic backgrounds throughout their life and increased energy expenditure. Single-nuclei RNA sequencing identified a dominant adipocyte population in brown adipose tissue (BAT) with a transcriptional signature of increased thermogenic activity, while white adipose tissue mass and beiging were reduced. Protection against diet-induced obesity was transferable to adult germ-free mice via exposure to natural microbiota, indicating an association with the timing of immune response induction rather than BAT developmental programming. However, protection against diet-induced obesity did not require type 2 immune signaling, as shown in STAT6 knockout mice. Rather, it was associated with increased levels of chemokines and monocytes in BAT in early life, pointing to a role of infiltrating myeloid cells. Indeed, CCR2-deficient mice with natural microbiota lacked the BAT transcriptional signature of increased thermogenic activity, increased energy expenditure, and protection against diet-induced obesity that wild-type mice with natural microbiota exhibited. The latter was restored upon injection with wild-type bone marrow. Taken together, these findings identify a novel microbiota‒immune‒adipose tissue axis that results in increased BAT thermogenesis and improved energy balance throughout life.

## Graphical abstract

**Figure:**
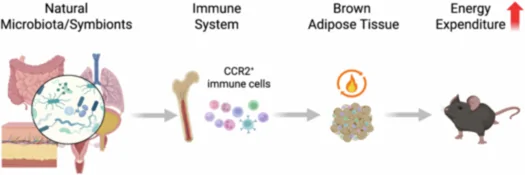

## Introduction

Obesity is recognized as a major public health challenge. The World Obesity Federation predicts that by 2030 there will be 1.13 billion obese adults worldwide, with an estimated 1.6 million premature deaths from obesity-related secondary diseases such as diabetes, cardiovascular disease, and cancer.^1^

Obesity is a multifactorial disorder shaped by numerous contributing factors, including changes in the gut microbiota.^2^ ^,^ ^3^ Studies in humans and in mouse models show that early-life disruption of the microbiota predisposes to obesity later in life^4^ and that high-fat diet and obesity have further negative effects on microbial communities.^5^ ^,^ ^6^ These alterations in microbiota affect energy harvest, inflammation, and metabolism, thereby perpetuating obesity and related comorbidities.^7^ ^,^ ^8^

It has also been proposed that bacterial taxa present before industrialization, which are still found in rural and hunter-gatherer populations,^9^ are progressively disappearing in modern-day humans.^10^ ^,^ ^11^ Similarly, mice that are used as preclinical models in the research laboratory lack many commensals that co-evolved with their free-living counterparts in the natural environment.^12^ ^,^ ^13^ This reduced microbial complexity limits the translational relevance of conventional mouse models.^14^ ^,^ ^15^

To restore complex natural microbiota in a preclinical model, we re-introduced natural microbiota from wild mice into laboratory mice by re-deriving C57BL/6NTac embryos in pseudo-pregnant wild mice.^16^ The pups acquired wild mouse microbiota and commensals at multiple body sites. They were used as founders of a colony of “wildling” mice, which retain the tractable genetics of C57BL/6 mice while harboring complex natural microbiota.^16^ The immune profile of wildling mice resembles that of microbe- and pathogen-experienced adult humans, thereby improving disease modeling.^16^ Of note, these naturalized immune responses were stable over years in the wildling mouse colony in a standard animal facility.^17^

While early-life microbiota disruption is known to increase obesity risk,^4^ we previously reported that early-life exposure to the natural microbiota protects against diet-induced obesity.^18^ Here, we demonstrate that this protection is lifelong and independent of diet and sex in different mouse strains. We further demonstrate that the natural microbiota alters the transcriptome of adipocytes in brown adipose tissue (BAT), thereby increasing energy expenditure. Importantly, this effect depended neither on the developmental age of the BAT nor on type 2 immune responses, which are known to induce thermogenesis. Rather, it required the recruitment of CCR2^+^ myeloid cells from the bone marrow. Thus, we define a novel microbiota‒immune‒adipose tissue axis that results in increased BAT thermogenesis and energy expenditure throughout life. These findings are of translational relevance because increased BAT activity is also associated with improved metabolic parameters in humans,^19^ opening the possibility that this can be achieved by modification of the microbiota.

## Materials and methods

### Mice

C57BL/6NTac wildling mice with microbiota, commensals, and pathogens from wild mice have been generated at NIDDK via transfer of C57BL/6NTac embryos into pseudopregnant wild mice^16^ and maintained as a colony in AALAC-accredited facilities at NIDDK.^17^ Specific-pathogen-free (SPF) C57BL/6NTac mice, hereafter referred to as lab mice, were purchased from Taconic Biosciences (Rensselaer, NY). Additionally, the following mice were used: BALB/cAnNTAC mice (Taconic Biosciences), hereafter referred to as Balb/c mice; B6.SJL-Ptprc^a^ Pepc^b^/BoyJ mice (The Jackson Laboratory), hereafter referred to as B6.SJL mice; C57BL/6:II-33–LacZ Gt mice^20^ (kindly provided by Dr. Jinfang Zhu, NIAID, NIH), hereafter referred to as IL-33 KO mice, B6.129 9Stat6Tm1 GRU/J mice (The Jackson Laboratory), hereafter referred to as STAT6 KO mice, and B6.129P2-*Ccr2* ^*tm1Mae*^ N10 (Taconic Biosciences), hereafter referred to as CCR2 KO mice; and CD45.1^+^ B6.SJL-Ptprc^a^/BoyAiTac mice (Taconic Biosciences), hereafter referred to as CD45.1^+^ C57BL/6 mice.

For high-fat diet challenge experiments, the mice were fed a rodent diet with 45 kcal% fat (Research Diet, Cat# D12451) or 60 kcal% fat (Research Diet, Cat# D12492) with choline or 45 kcal% fat without choline (Research Diet, Cat# D05010402).

Experiments were performed following the Guide for the Care and Use of Laboratory Animals under an animal study protocol approved by the NIDDK Animal Care and Use Committee.

### Transfer of natural microbiota and commensals from wildling mice to mice with different genetic backgrounds

The wildling Balb/c colony was created by gavaging ileocecal content from wildling C57BL/6NTac mice into germ-free Balb/c mice. The weaned offspring of wilding Balb/c mice were co-housed with wildling C57BL/6NTac mice for four weeks in large cages. Wildling B6.SJL, IL-33 KO, STAT6 KO, or CCR2 KO colonies were created by co-housing the respective specific pathogen-free (SPF) mice with female wildling C57BL/6NTac mice at a ratio of 1:3 for four weeks. The resulting wildling B6.SJL, wildling IL-33 KO, wildling STAT6 KO, and wildling CCR2 KO were founders of separately maintained colonies and received bedding from wildling C57BL/6NTac mice at each cage change. Experiments were performed with the offspring unless otherwise stated.

### DNA extraction, 16S rRNA gene sequencing, and bioinformatic analysis

DNA was extracted from cryopreserved mouse fecal pellets using the MagAttract PowerMicrobiome DNA/RNA Kit (Qiagen) and fully automated Eppendorf liquid-handling robots (epMotion 5075 and epMotion 5073) according to the manufacturer's instructions. 16S rRNA gene sequencing was performed with primers targeting the V4 region (515F–806R). Libraries were sequenced on the Illumina MiSeq platform, generating 2 × 300-bp paired-end reads.

16S rRNA gene (V4) sequence data were processed with DADA2 (v1.14.0) to infer sequence variants.^21^ Data integration was performed in R (v3.5.0) using the JAMS package (v1.9.4).^22^ Principal component analysis (PCA) was conducted in JAMS (v1.9.6), and heat maps were generated using the ComplexHeatmap package (v2.6.2) via the plot_relabund_heatmap function in JAMS.

### Pathogen screening

Blood samples were tested for pathogens using Opti-Spot Cards and for mouse hepatitis virus using the Mouse Comprehensive Serology Panel (IDEXX BioAnalytics). Fur swaps and fecal pellets were tested by real-time PCR (EDx/Opti-XXPress Prevalent Panel (IDEXX BioAnalytics), with additional assays for pathogens, including *Tritrichomonas muris*, *Pseudomonas aeruginosa*, and *Giardia muris*).

### Indirect calorimetry and body composition analysis

An Oxymax/CLAMS system (Columbus Instruments) was used to measure total energy expenditure, total activity (beam breaks), RER (respiratory exchange ratio, which is the ratio between O_2_ consumed and CO_2_ produced), and food intake. The mice were single-housed per cage for 5‒7 d at 22 °C or 30 °C ambient temperature as indicated. Measurements were taken every 7 minutes after 2 d of acclimation.

Body composition (fat mass and lean mass) was measured using an Echo MRI 3-in-1 (Echo Medical Systems).

### Bone marrow transfer

Bone marrow donors were euthanized according to NIH protocol. The tibias and femurs were removed and placed in cold DPBS on ice. After snipping off the top of the femur and tibia, the bone marrow was flushed out into 50 mL tubes using a syringe with a 25 G needle filled with RPMI cell culture medium (Corning) containing 10% FBS (Sigma-Aldrich) and then centrifuged at 200 × g for 5 minutes at 4 °C. The cells were resuspended in red blood cell lysis buffer (Boston BioProducts), incubated at room temperature for 3 minutes, and then washed with RPMI containing 10% FBS. The suspension was filtered through 40-μm cell strainers (BD Falcon) and centrifuged at 200 × g for 5 minutes at 4 °C. The cells were resuspended in 1x DPBS (Corning) and counted with the Revvity Cellometer™ Ascend™ automated cell counter. Four to twenty million wildling bone marrow cells were transferred by intraperitoneal injection into 7–10-day-old pups of wildling CCR2 KO mice or lab CCR2 KO mice.

### Histology

Mice were euthanized according to NIH protocol. BAT was harvested and fixed in neutral-buffered 10% formalin solution for 48 hours and then transferred to 70% ethanol. Tissue embedding in paraffin, H&E staining of 3 μm sections, and scanning of the slides were performed at Histoserv (Gaithersburg, MD). The digital images were analyzed with Qupath, version 0.5.1. Lipid droplets were quantified in adipocytes in five random fields at 20× magnification for each mouse using ImageJ software.

### Isolation of cell nuclei from BAT for single-nuclei RNA sequencing or quantitative real-time PCR

Interscapular BAT was harvested and either processed directly for snRNA-seq or snap-frozen in liquid nitrogen and stored at −80 °C for later use for quantitative real-time PCR. Fresh or frozen BAT was minced and then homogenized on ice with nuclear preparation buffer (10 mM HEPES, 1.5 mM MgCl_2_, 10 mM KCl, 250 mM sucrose, 0.05% IGEPAL CA-630, 0.2 mM DTT), supplemented with protease inhibitor cocktail (Roche) and 0.02 units/μl RNAse-SUPERase•In™ RNase RNase Inhibitor (Invitrogen). The BAT homogenate was filtered through a 70 μm (fresh BAT) or 40 μm (frozen BAT) cell strainer and centrifuged at 1000 × g for 10 minutes at 4 °C. After the supernatant was removed, the nuclei pellets were resuspended in nuclear preparation buffer with 1 unit/μl (fresh BAT) or 0.02 units/μl (frozen BAT) of RNAse-SUPERase•In™ RNase Inhibitor (Invitrogen) and protease inhibitor cocktail (Roche) and centrifuged under the same conditions (fresh BAT) or at 600 × g for 7 minutes at 4 °C (frozen BAT). The pellets were then resuspended in nuclear resuspension buffer (1% BSA in DPBS, 2 mM MgCl_2_) with 0.04 units/μl (fresh BAT) or 0.02 units/μl (frozen BAT) RNAse-SUPERase•In™ RNase Inhibitor (Invitrogen) and protease inhibitor cocktail (Roche).

For the snRNAseq experiments, the nuclear resuspension buffer was supplemented with DAPI (BioLegend) to label the nuclei for 15 minutes on ice. Nuclei (2N) were sorted using a BD FACSAria™ Fusion instrument. The sorted nuclei were washed twice, resuspended in 1× PBS solution, and counted using a Cellometer Auto 2000 cell viability counter (Nexcelom Bioscience). The 10× Genomics Chromium controller was loaded with 16,000 nuclei/sample to generate droplets, aiming to capture 10,000 nuclei per well. Following reverse transcription, cDNA was purified using Dynabeads™ MyOne™ SILANE (PN-2000048) and amplified. The amplified cDNA was purified with SPRIselect beads (Beckman Coulter). A 3ʹ gene expression library was prepared with the Chromium Next GEM Single Cell 3ʹ Reagent Kit v3.1 (10X Genomics) according to the manufacturer's instructions. The quantity and quality of each library were determined using a TapeStation (Agilent Technologies) and DS-11 FX+ (DeNovix). All libraries were pooled and sequenced on the NovaSeq6000 (Illumina) with 28 bp for read 1 and 90 bp for read 2.

To generate RNA for cDNA synthesis and quantitative real-time PCR (qPCR), the nuclear resuspension buffer was removed from the cell pellets after centrifugation. The cell pellets were resuspended in 100 μl of lysis solution provided with the RNAqueous™-Micro Total RNA Isolation Kit, Invitrogen™ (Invitrogen). RNA was extracted with RNAqueous™-Micro Total RNA Isolation Kit, Invitrogen™ (Invitrogen) according to the manufacturer’s protocol. cDNA was synthesized, and qPCR was performed as described below for WAT tissue.

### RNA extraction from WAT and qPCR

Inguinal WAT tissue was harvested, snap-frozen in liquid nitrogen, and stored at −80 °C. RNA was extracted using TRIzol™ (Thermo Fisher Scientific) and chloroform. After centrifugation (12,000 × g), the RNA was precipitated with isopropanol, washed with 75% ethanol, and dissolved. cDNA was synthesized from 1 μg of total RNA treated with DNase I (Thermo Fisher Scientific), using the Maxima First Strand cDNA Synthesis Kit for RT-qPCR (Thermo Fisher Scientific) according to the manufacturer's protocol. Real-time PCR was conducted using the Applied Biosystems™ Power Syber™ Green PCR mix (Thermo Fisher Scientific) and the QuantStudio 6 pro Applied Biosystems™ (Thermo Fisher Scientific). Normalization was performed against *Actb, 18S,* or *Rpl7,* and relative gene expression was calculated using the 2^−ΔΔCT^ method. The primer sequences used are provided in Table S1.

### Processing, integration, and analysis of single-nuclei RNA sequencing data

SnRNA-seq data were obtained from the BAT of two lab and two wildling mice (7–8-week-old male mice) that had been housed at 23 °C and from two lab and two wilding mice that had been housed at 27 °C–28 °C ambient temperature. Ambient RNA was removed using CellBender^23^ (version 0.2.0), and doublets were detected and removed using a combination of scores calculated via *scDblFinder* ^24^ (version 1.2.0) and *scds* ^25^ (version 1.4.0). Low-quality cells were filtered using cutoffs of 400 UMIs/cell and 10% mitochondrial transcripts. This resulted in the exclusion of data from BAT of one wildling mouse housed at 27 °C–28 °C ambient temperature from further analysis.

The cells were analyzed with Seurat^26^ (version 4.1.1), using *SCTransform* ^27^ to normalize the data and using *CCA* to integrate with 3000 variable genes. To subcluster the cells, they were split into “adipocytes” and “svf” based on previously identified marker genes. The relevant cells were subseted and reintegrated using 1500 variable genes. Any subclustered populations with high doublet scores as calculated by *scDblFinder* and *scds* were removed, as were any with high mitochondrial gene content. Cell clusters and subclusters were annotated via mapping to published mouse adipose sNuc-seq data.^28^ Ambient RNA was removed using CellBender^23^ (version 0.2.0), and doublets were detected and removed using a combination of scores calculated via *scDblFinder* ^24^ (version 1.2.0) and *scds* ^25^ (version 1.4.0). Low-quality cells were filtered using cutoffs of 400 UMIs/cell and 10% mitochondrial transcripts. The cells were analyzed with Seurat^26^ (version 4.1.1), using *SCTransform* ^27^ to normalize the data, and using *CCA* to integrate with 3000 variable genes. To subcluster the cells, they were split into “adipocytes” and “svf” based on previously identified marker genes. The relevant cells were subseted and reintegrated using 1500 variable genes. Any subclustered populations with high doublet scores as calculated by *scDblFinder* and *scds* were removed, as were any with high mitochondrial gene content. Cell clusters and subclusters were annotated via mapping to published mouse adipose sNuc-seq data.^28^

To calculate pseudocounts, adipocytes were split by sample, and counts for each gene were summed. Differential expression analysis was performed on the pseudo counts using edgeR^29^ (version 3.30.3).

Gene set enrichment analysis was performed on the results using *fgsea* (version 1.21.2)^30^ on Reactome pathways.^31^ To select the most enriched pathways for presentation, pathways were filtered to remove any less significant pathway with more than 85% gene similarity to a more enriched pathway.

### Quantitation of cytokines in BAT

Interscapular BAT was excised, snap-frozen in liquid nitrogen, and stored at −80 °C until use. Frozen BAT was homogenized with RIPA lysis and extraction buffer using 5 mm stainless steel grinding beads (Thomas Scientific) and the TissueLyser LT (Qiagen). The homogenates were incubated on ice for 1  hour and then centrifuged at 20,000 × g for 15 minutes at 4 °C. The liquid phase was transferred to a new tube and centrifuged again under the same conditions. The supernatant was transferred to a new tube, and the protein concentration was determined using the Pierce BCA Protein Assay Kit (Thermo Fisher Scientific) according to the manufacturer's protocol.

One hundred milligrams of protein from each tissue was used to measure the cytokine concentration with the V-PLEX Mouse Cytokine 19-Plex Kit and the V-PLEX Custom Mouse Biomarkers Proinflammatory Panel 1 (Mesoscale) following the manufacturer's protocol. The relative abundance of each cytokine per milligram of tissue was calculated.

### Flow cytometry analysis of immune cells from BAT

The mice were euthanized according to NIH protocol and postmortem perfused with 1× PBS via cardiac puncture. BAT was excised, incubated in RPMI containing 10% FBS on ice, then minced and digested in RPMI containing 2% FBS supplemented with collagenase type IV (1 mg/ml; Worthington Biochemical Corp) or Liberase™ TL (0.25 mg/ml; Roche) at 37 °C with gentle shaking for 45 minutes. The digested tissue was filtered through a 40-μm cell strainer (BD Falcon) into a new tube, RPMI containing 5% FBS was added, and the cell suspension was centrifuged at 2000 rpm for 5 minutes at 4 °C. The cell pellet was resuspended in red blood cell lysis buffer and incubated at room temperature for 3 minutes, then resuspended in RPMI containing 5% FBS and centrifuged at 2000  rpm for 5 minutes at 4 °C. The supernatant was discarded, and the cell pellet was resuspended in RPMI containing 2% FBS. The cells were counted using either a Cellometer Auto 2000 cell viability counter (Nexcelom Bioscience) or a Revvity Cellometer™ Ascend™ automated cell counter.

The cell suspensions were incubated with CD16/32 mAb (Clone: 2.4G2, BD Biosciences, 1:200 dilution) to block Fc-receptors. The cell surface antigens were stained at 4 °C for 30  min using a mixture of the LIVE/DEAD fixable blue dead cell stain kit (Thermo Fisher Scientific, 1:500 dilution) and combinations of the following antibodies that target B220/CD45R (Clone RA306B2 conjugated to BV510), RORγt (Clone Q31-378 conjugated to BV650), GATA3 (Clone L50-823 conjugated to BB700), CD4 (Clone GK1.5 conjugated to BV496), CD45.2 (Clone 104 conjugated to BUV395), CD8a (Clone 53-6.7 conjugated to BUV805), Ly-6C (Clone AL-21 conjugated to FITC), Ly-6G (Clone 1A8-BV421), NK1.1 (Clone PK136 conjugated to PE-CF594), CD127 (Clone SB/199 conjugated to BV711), CD11b (Clone M1/70 conjugated to BV750), CD16/32 (Clone 2.4G2) (all from BD Biosciences), CD3 (Clone 17A2 conjugated to Alexa Fluor 700), Ly-6C Antibody (Clone HK1.4 conjugated to FITC), F4/80 (Clone BM8 conjugated to Alexa Fluor 647), T-bet (Clone 4B10 conjugated to PE/Cyanine7), CD3ε (Clone 145-2C11 conjugated to BV711), CD45.1 (Clone A20 conjugated to APC) or CD90.2 (Thy1.2) (Clone 30-H12 conjugated to Alexa Fluor 700) (all from Biolegend). The stained samples were analyzed on a BD FACS Symphony flow cytometer using FACS Diva Version 6.1.3 (BD Biosciences). The data were analyzed with FlowJo software version 10.9.0 (Tree Star).

### Statistics

Mann‒Whitney tests were used to compare values between two independent groups, with Benjamini‒Hochberg false discovery rate (FDR) correction if multiple features were assessed simultaneously. The Kruskal‒Wallis test or Brown‒Forsythe and Welch tests with Benjamini–Hochberg FDR correction were applied to more than two groups. PERMANOVA and F-model scores were used to compare groups in dimensional reduction plots. Statistical significance was indicated as *p < 0.05, **p < 0.01, *** *p* < 0.001, **** *p* < 0.0001.

## Results

### Wildling mice gain less body weight than conventional lab mice, irrespective of diet, sex, genetics, and age

**Figure 1.**
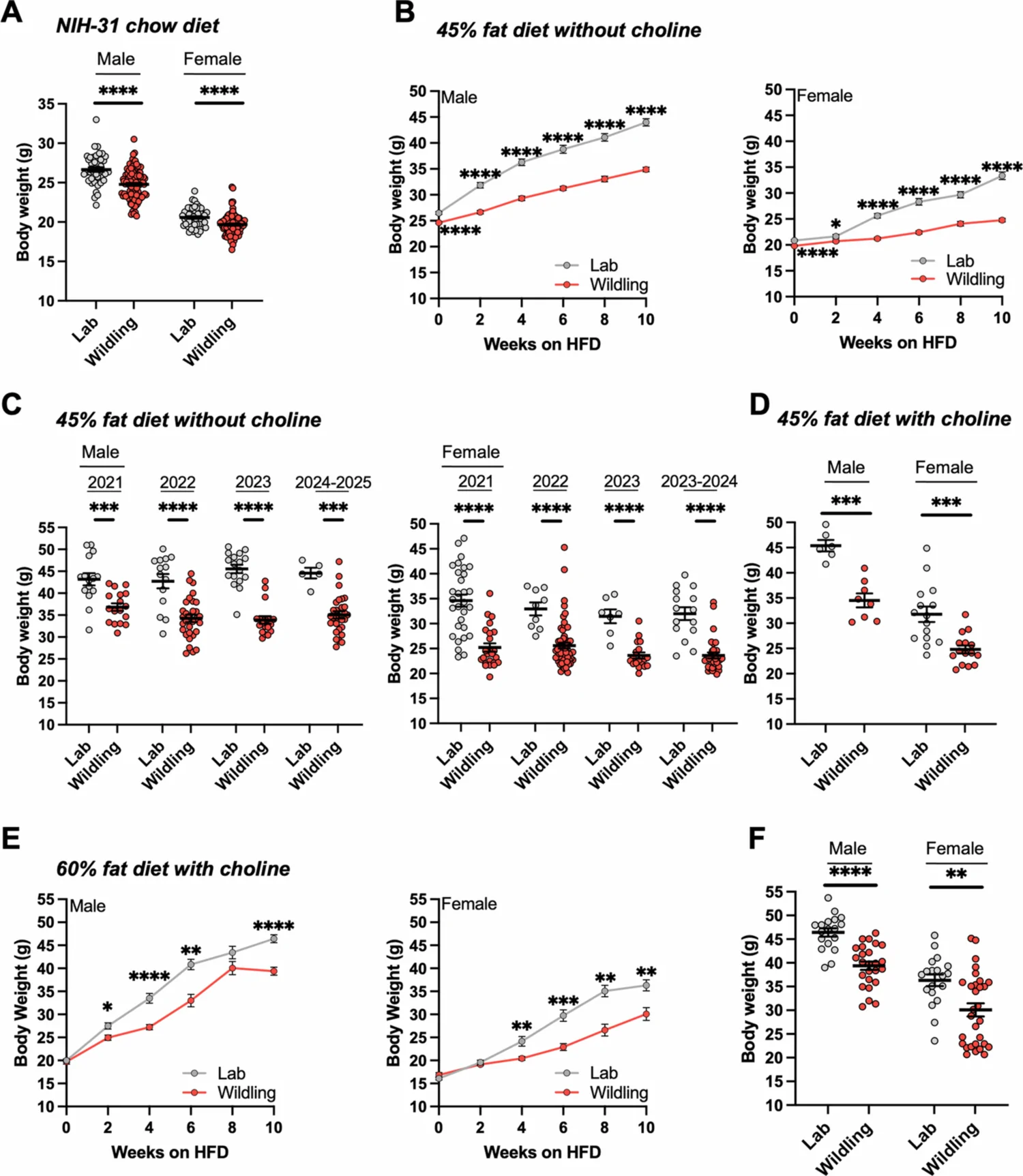
Wildling mice gain less body weight than lab mice, irrespective of diet and sex. (A) Weight of C57BL/6NTac wildling mice and conventional C57BL/6NTac lab mice on NIH-31 chow diet (85 females, 104 male wildling mice, 50 females, 43 male lab mice). (B and C) Weight of C57BL/6NTac wildling mice and conventional C57BL/6NTac lab mice during (B) and after (C) a 10-week course of a choline-deficient 45% fat diet. The data are combined in (B) and stratified by year in (C). Wildling mice: 134 females, 95 male; lab mice: 61 females, 51 males. (D) Weight of C57BL/6NTac wildling mice and conventional C57BL/6NTac lab mice after a 45% fat diet with choline (15 female, 8 male wildling mice, 15 female, and 6 male lab mice). (E and F) Weight of C57BL/6NTac wildling mice and conventional C57BL/6NTac lab mice during (E) and after (F) a 10-week course of a 60% fat diet with choline (30 female, 25 male wildling mice; 19 female, 18 male lab mice). ****p < 0.0001, ***p < 0.001, **p < 0.01, *p < 0.05 (Mann‒Whitney test). Benjamini‒Hochberg FDR correction was applied in B, E. The mean and SEM are shown. HFD, high-fat diet.

We previously published that C57BL/6NTac mice with natural microbiota and pathogens from wild mice (wildling mice) are protected from excessive weight gain when fed a choline-deficient, 45% fat diet from week 10 to week 20 of life.^18^ We now report that male and female wildling mice also exhibited lower body weight than their conventional C57BL/6NTac lab mouse counterparts on an NIH-31 chow diet (Figure 1A) and that the observed protection against high-fat diet-induced obesity was preserved in the wildling mouse colony for more than 5 years (Figure 1B, C). We additionally challenged the mice with choline-containing 45% and 60% fat diets and confirmed that wildling mice gained significantly less weight than lab mice (Figure 1D–F).

**Figure 2.**
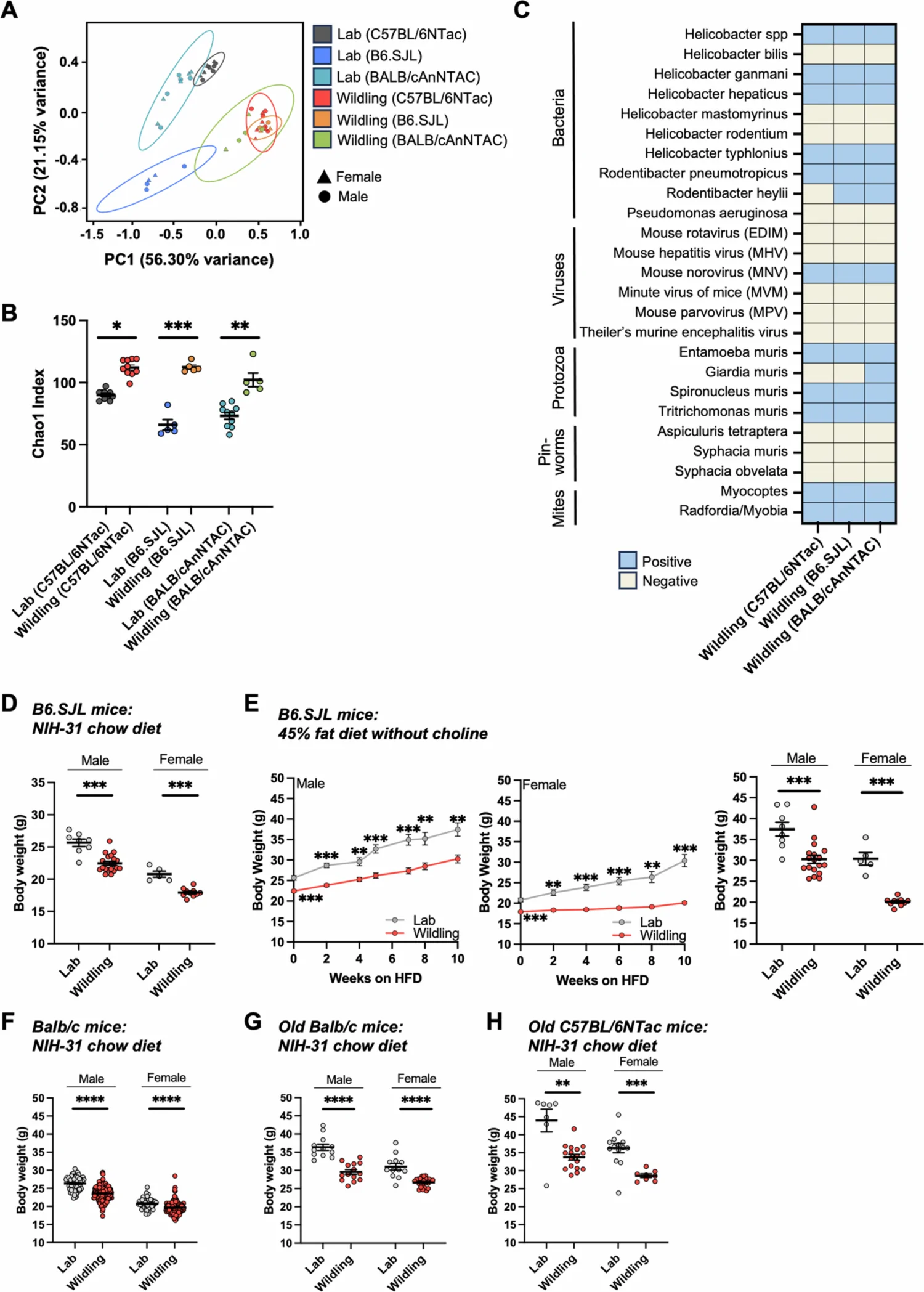
Wild-derived microbiota confer lifelong protection against diet-induced obesity in mice of various genetic backgrounds. (A–C) Wildling mice on the B6.SJL and Balb/c background were created as independent colonies whose founders were B6.SJL and Balb/c lab mice co-housed for four weeks with C57BL/6NTac wildling mice. 16S rRNA gene profiling was performed on the fecal pellets of 10 C57BL/6NTac lab mice, 10 Balb/c lab mice, and 5 B6.SJL lab mice, 10 C57BL/6NTac wildling mice, 5 B6.SJL wildling mice and 5 Balb/c wildling mice. (A) PCA of last known taxa (LKT), normalized to the number of bases for analysis in the sample and log2 transformed, with the Bray dissimilarity index applied. *p* < 1e−04 (PERMANOVA); F−Model = 16.3616. (B) Chao 1 ⍺-diversity index. * *p* < 0.05, ** *p* < 0.01, *** *p* < 0.001 (Kruskall‒Wallis test with Benjamini‒Hochberg FDR correction). C, Results of pathogen screening by real-time PCR on fecal pellets and skin swabs (IDEXX BioAnalytics) using pooled samples from 5 mice for each group. (D and E) Weight of BL6.SJL wildling mice and BL6.SJL lab mice on NIH-31 chow diet (D, 10 female, 19 male wildling mice; 5 female, 8 male lab mice) and during and after a 10-week course of a choline-deficient 45% fat diet (E, 10 female and 18 male wildling mice; 5 female and 8 male lab mice). (F–H) Weight of Balb/cAnNTac wildling and Balb/cAnNTac lab mice at weeks 8–10 of age (F, 186 female and 158 male wildling mice; 53 female and 74 male lab mice), and at weeks 57–67 of age (G, 25 female, 14 male wildling mice; 12 female, 12 male lab mice) and C57BL/6NTac wildling and C57BL/6NTac lab mice at week 60 of age (H, 8 female and 17 male wildling mice; 14 female and 7 male lab mice) on NIH-31 chow diet. ****p < 0.0001, ***p < 0.001, **p < 0.01 (Mann‒Whitney test, Benjamini‒Hochberg FDR correction was applied in E). The means and SEM are shown. HFD, high-fat diet.

To exclude a genetic effect of the C57BL/6NTac strain, we created wildling mice on the Balb/c background by gavaging ileocecal content from wildling C57BL/6NTac mice into germ-free Balb/c mice and co-housing the wilding Balb/c off-spring with wildling C57BL/6NTac mice. A wildling C57BL/6.SJL colony was established by co-housing SPF lab C57BL/6.SJL with C57BL/6NTac wildling mice. Wildling Balb/c and wildling C57BL/6.SJL colonies were maintained independently, with continued exposure to bedding from the wildling C57BL/6NTac mice. Profiling of fecal pellets via 16S rRNA gene sequencing confirmed that C57BL/6.SJL and Balb/c mice acquired microbiota from wildling C57BL/6NTac mice (Figure 2A, Figure 2B). Principal component analysis (PCA; Figure 2A) revealed that all wildling samples clustered closely together and were separate from those of conventional lab mice. Although not all taxa were transferred to Balb/c mice (Supplementary Figure S1), microbiota diversity was significantly higher in wildling than lab mice with the corresponding B6.SJL or Balb/c backgrounds, respectively (Figure 2B). Additionally, all the tested pathogens were transferred (Figure 2C). Importantly, male and female wildling C57BL/6.SJL mice gained significantly less weight than lab C57BL/6.SJL mice when placed on either NIH-31 chow diet (Figure 2D) or choline-deficient 45% fat diet (Figure 2E). Similarly, male and female wildling Balb/c mice gained significantly less weight than lab Balb/c mice on NIH-31 chow diet (Figure 2F), and the differential weight gain extended to old age in wildling Balb/c and C57BL/6NTac mice (57–68 weeks of age, Figure 2G, H). Together, these results indicate that four weeks of cohousing is sufficient for transferring the metabolic phenotype to conventional lab mice.

Thus, wildling mice gained less body weight compared to lab mice across diets, and this effect was independent of sex, mouse genetic strain and age.

### Wildling mice have higher energy expenditure than conventional lab mice

**Figure 3.**
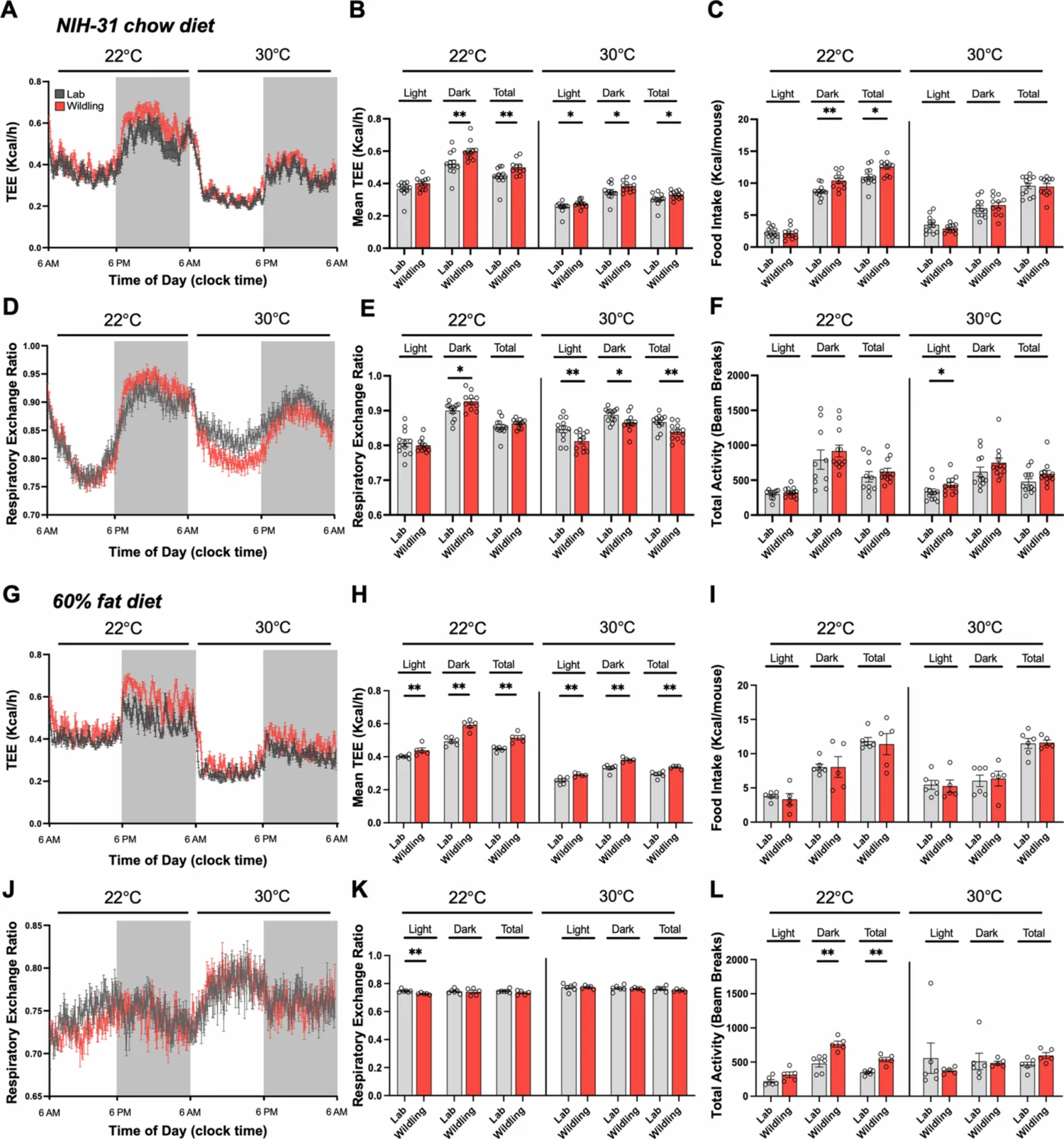
Wildling mice have higher energy expenditure than conventional lab mice. Male C57BL/6NTac wildling and lab mice were individually housed in metabolic cages at the indicated ambient temperature after being fed NIH-31 chow diet (A-F, 11–12 wildling and 12 lab mice; 10–17 weeks of age) or NIH-31 chow diet followed by one week of 60% fat diet (G–L, 5 wildling and 6 lab mice; 11–13 weeks of age). The raw data were acquired over 24 h at 22 °C and 24 h at 30 °C. The bar graphs indicate the mean plus SEM of the data per light or dark cycle. Total, 24 hours. (A, B, G, H) total energy expenditure (TEE); (C and I) food intake; (D, E, J, K) respiratory exchange ratio (F, L) total activity. *p < 0.05, **p < 0.01 (Mann‒Whitney test). The means and SEM are shown.

To investigate the mechanisms of protection against diet-induced obesity, we performed indirect calorimetry. Male wildling mice fed with a chow diet had significantly higher total energy expenditure (TEE) than lab mice on the same C57BL/6NTac background at an ambient temperature of 22 °C, which is standard in animal facilities (Figure 3A, B). Female wildling mice, which are smaller than male wildling mice (Figure 1A), showed the same trend (not shown). Wildling mice consumed significantly more food than lab mice (Figure 3C) and had an increased respiratory exchange ratio (RER) during the dark cycle, which is the active phase for mice (Figure 3D, E), without any difference in physical activity (Figure 3F). These findings indicate that the natural microbiota increase energy expenditure and that the mice increase their food consumption to meet their energy demands.

Next, we housed the mice for 24 hours at 30 °C, an ambient temperature that represents thermoneutrality and reduces non-shivering thermogenesis.^32^ As expected, both the wildling and lab mice showed a significant reduction in energy expenditure (Figure 3A, B). Despite this reduction, wildling mice still displayed slightly higher energy expenditure than lab mice (Figure 3A, B), without any significant difference in food intake and physical activity (Figure 3C, F). However, at 30 °C, wildling mice had a reduced RER compared to lab mice (Figure 3D, E), suggesting relatively increased lipid oxidation and reduced carbohydrate utilization.^33^ ^,^ ^34^

We next assessed the metabolic parameters of the mice after one week on a 60% fat diet. Again, male wildling mice showed significantly higher energy expenditure than male lab mice (Figure 3G, H). Both groups of mice displayed a significant reduction in energy expenditure when the ambient temperature was increased to 30 °C, with wildling mice still expending more energy than lab mice (Figure 3G, H). There was no difference in food intake between wildling mice and lab mice (Figure 3I) and no difference in RER during the active phase (Figure 3J, K), even though wildling mice were more active than lab mice at 22 °C (Figure 3L). Because wildling mice consumed the same amount of food as lab mice at increased ambient temperature and when fed with HFD (Figure 3C, I) but still displayed higher energy expenditure (Figure 3B, H), the thermal effect of food was excluded. Taken together, these results demonstrate that the wild-derived microbiota induce a unique metabolic phenotype with increased energy expenditure in mice, which is caused neither by increased activity nor by a thermal effect of the consumed food.

### Wildling mice exhibit altered BAT morphology and an expanded adipocyte population with a transcriptomic profile of increased thermogenesis

**Figure 4.**
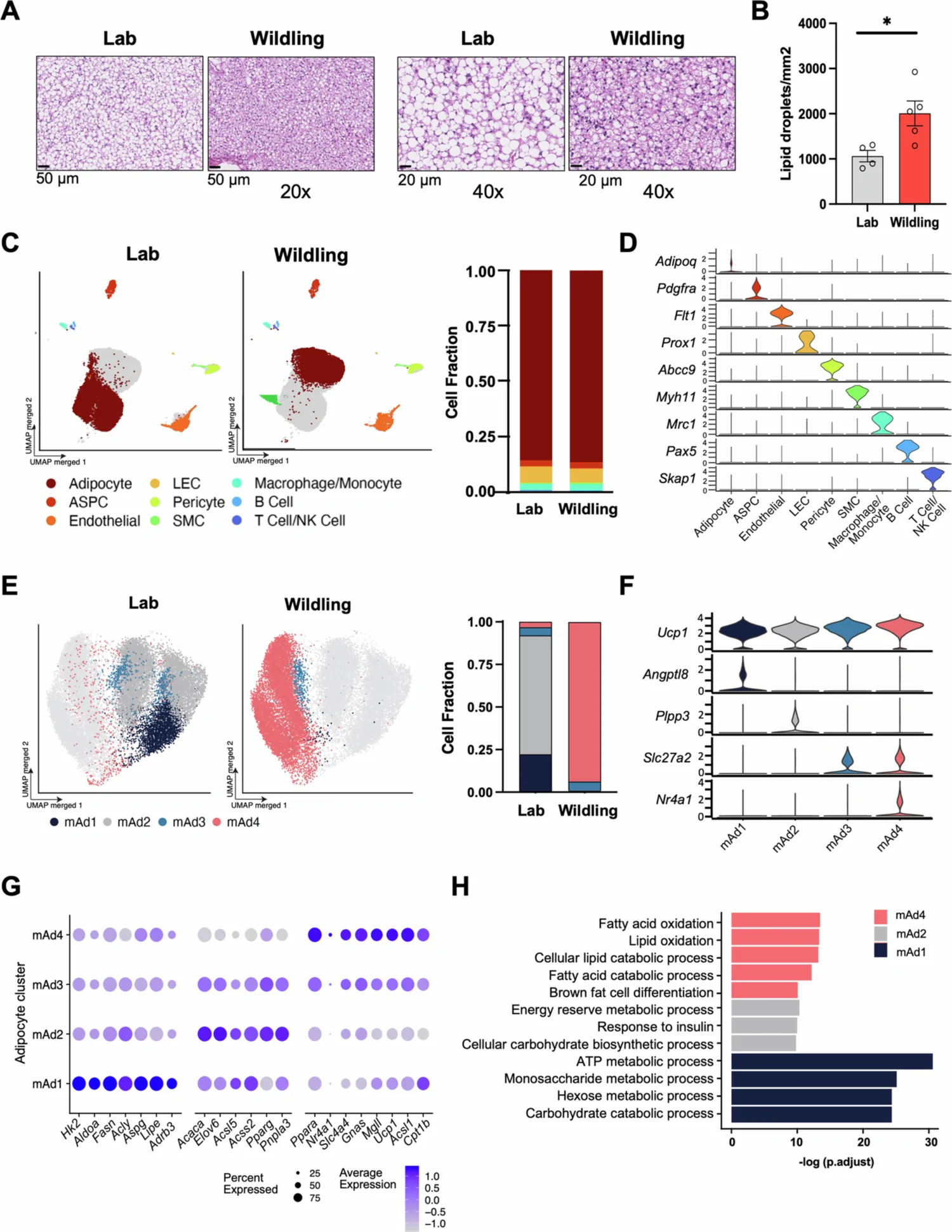
Wildling mice have an altered BAT morphology and brown adipocyte transcriptomes. (A) Histology (hematoxylin and eosin stain) of the interscapular BAT of C57BL/6NTac wildling and C57BL6/NTac lab mice, representative of 12 mice per group from two independent experiments. Original magnification ×20 or ×40; scale bars, 20 or 50 µm. (B) Quantification of lipid droplets in BAT (5sections per mouse, 5 wildling, and 4 lab mice). (C) Uniform manifold approximation and projection (UMAP) plot of an integrated analysis of single-nuclei RNA sequencing data of interscapular BAT from 2 wildling and 2 lab mice housed at room temperature (22 °C). The highlighted nuclei are from mice housed at room temperature (lab, 14,418 nuclei; wildling, 11,838 nuclei). Nuclei are colored by cell type; gray indicates nuclei from other conditions. The bar graphs show the relative size of the identified populations. (D) Violin plots showing the expression of selected marker genes. (E) UMAP plot of adipocyte nuclei from BAT of lab and wildling mice housed at room temperature (22 °C) (lab, 12,460 nuclei; wildling, 10,188 nuclei). Nuclei in the plotted condition are colored by cell type; light gray cells in the left UMAP represent cells from wildling mice, and light gray cells in the right UMAP represent cells from lab mice. The bar graphs show the relative size of the four identified populations (mAd1 to mAd4). (F) Violin plots of *Ucp1* as a marker gene for thermogenic adipocytes in addition to marker genes for each identified adipocyte cluster in BAT of lab and wildling mice housed at 22 °C. (G and H) Dot plot showing the expression of selected cluster marker genes (G) and gene set enrichment analysis showing enriched pathways (H) in the indicated adipocyte clusters. ASPCs, adipocyte stem and progenitor cells; LECs, lymphatic endothelial cells; SMCs, smooth muscle cells.

Hematoxylin and eosin staining revealed an increased density of multilocular adipocytes with higher number of lipid droplets and more obvious cell boundaries in BAT of wildling mice than lab mice (Figure 4A, B), a morphology that is associated with increased BAT activity. To evaluate gene expression in BAT of lab and wildling mice, we performed single-nuclei RNA sequencing of flow cytometry-sorted nuclei from BAT of two lab and two wildling mice on NIH-31 chow diet. Integrated analysis of the transcriptomes of 48,204 nuclei from BAT of lab and wildling mice identified adipocytes, adipocyte-stem and progenitor cells, endothelial cells, pericytes, smooth muscle cells, macrophages and monocytes, B cells, T cells, and NK cells by expression of cell type-specific marker genes (Figure 4C, D).

More than 80% of the nuclei were annotated as adipocytes, and the proportion of adipocytes in BAT from wildling mice was similar to that in BAT from lab mice (Figure 4C). However, the adipocyte nuclei from wildling mice mapped to a distinct region in the integrated UMAP relative to those from lab mice (Figure 4C), indicating qualitative differences in the adipocyte state between the two groups. To gain further insight, we re-clustered the adipocytes and identified four distinct subclusters, mAd1, mAd2, mAd3, and mAd4 (Figure 4E, F). The mAd4 cluster accounted for more than 95% of the adipocyte nuclei in wildling mice, whereas the mAd1 and mAd2 clusters were almost exclusively present in lab mice (Figure 4E, the nuclei of the respective other groups are shown in light gray in each UMAP). The smallest cluster (mAd3) was found in both groups of mice (Figure 4E).

Thus, BAT of wildling mice contained a large, homogeneous population of brown adipocytes (mAd4) that was not found in BAT of lab mice. This mAd4 cluster expressed genes associated with fatty acid oxidation and thermogenesis, such as *Ucp1*, *Ppara* and *Gnas* (Figure 4F, G).^35^ ^,^ ^36^ mAd4 additionally expressed *Nr4a1,* which encodes Nur77, a known regulator in activated BAT,^37^ and shared with mAd3 the expression of *Slc27a2* (Figure 4F), which encodes a protein that is involved in the uptake of long-chain fatty acids in support of heat production in BAT.^38^ Gene set enrichment analysis identified as activated pathways fatty acid and lipid oxidation, catabolic processes, and brown adipocyte differentiation in the mAd4 adipocyte cluster (Figure 4H).

In contrast, adipocyte clusters mAd1 and mAd2 expressed the marker genes *Angptl8* and *Plpp3*, respectively (Figure 4F), which both encode proteins involved in lipid metabolism.^39^ ^,^ ^40^ In addition, these clusters exhibited increased expression of the transcripts encoding key enzymes involved in *de novo* lipogenesis, including *Fasn*, *Acly*, and *Acaca*, which encode fatty acid synthase, ATP-citrate lyase, and Acetyl-CoA carboxylase alpha, respectively. The transcript levels of *Elovl6* were also elevated. *Elovl6* encodes elongation of very long-chain fatty acid protein 6, a key regulator of lipid metabolism that has been implicated as a metabolic checkpoint in insulin sensitivity, obesity, and cancer progression (Figure 4G).^35^ ^,^ ^41^ ^,^ ^42^ Of note, the marked differential clustering of brown adipocyte nuclei from lab and wildling mice in the UMAP was much reduced when the mice were housed at increased ambient temperature (Supplementary Figure S2).

Collectively, these results demonstrate that the natural microbiota altered the transcriptional program of adipocytes in BAT, resulting in a dominant major adipocyte population with increased expression of genes associated with thermogenic activity and enrichment in lipid catabolic pathways. Differential gene expression existed at baseline, without any high-fat diet challenge, in wildling and lab mice and was reduced when the mice were housed at thermoneutrality.

### Natural microbiota reduce the expression of thermogenic genes in inguinal white adipose tissue

Next, we evaluated the potential contribution of beiging of white adipose tissue (WAT) to the metabolic phenotype of the wildling mice. Wildling mice had less inguinal WAT (iWAT) mass than lab mice (Supplementary Figure S3A), with significantly lower expression of *Lep* transcripts (Supplementary Figure S3B) and lower serum levels of the corresponding protein leptin (Supplementary Figure S3C). Histologic analysis demonstrated smaller lipid droplets in iWAT of wildling mice compared to lab mice, resulting in a significantly higher number of lipid droplets per area (Supplementary Figure S3CD). Importantly, the expression of thermogenic genes was reduced in iWAT wildling mice compared to lab mice (Supplementary Figure S3E), implying that there was no increased beige adipocyte activity and/or browning in wildling iWAT. Additionally, there was no differential expression of *Alpl*, *Ckb*, and *Gatm* transcripts (Supplementary Figure S3F), which indicates that a recently discovered UCP-1-independent pathway of thermogenesis, the futile creatine cycle,^43^ ^,^ ^44^ was not activated. Taken together, these results demonstrate that the metabolic phenotype of wildling mice does not involve beiging of iWAT.

### Protection against diet-induced obesity depends on immune response induction rather than BAT developmental programming

**Figure 5.**
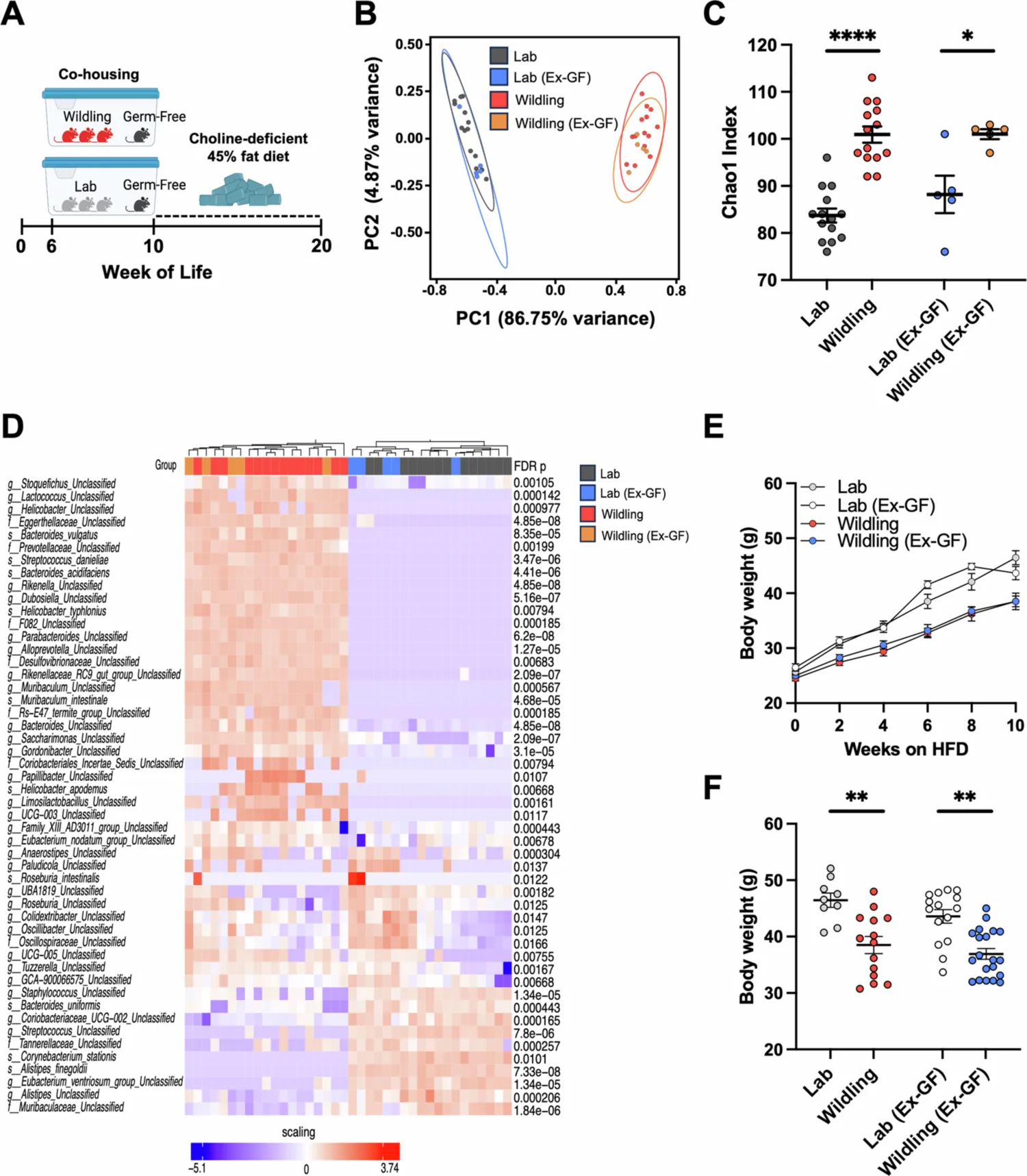
The wildlings' protection against diet-induced obesity can be transferred to adult germ-free mice. (A) Experimental design. Male germ-free (GF) C57BL/6NTac mice were co-housed with lab mice or wildling mice from week 6 to 10 of life and then fed a 10-week course of choline-deficient 45% fat diet. (B–D) Analysis of the gut microbial composition of wildling mice and lab mice (14 per group) and co-housed former GF mice (ex-GF, 5 per group). 16S rRNA gene profiling was performed on the fecal pellets of male C57BL/6NTac mice that had been purchased in GF status and co-housed with lab or wildling mice from week 6 to 10 of life. Analysis was performed prior to the fat diet. (B) PCA of last known taxa (LKT), normalized to the number of bases for analysis in the sample and log2 transformed, with the Bray dissimilarity index applied. The ellipses indicate 90% confidence regions. *p* < 0.0001 (PERMANOVA); F-Model = 18.8205. (C) Chao 1 ⍺-diversity index. *p < 0.05, ****p < 0.0001 (Mann‒Whitney test). The means and SEM are shown. (D) LKT-level heat map generated by unsupervised hierarchical clustering, showing the top 50 most variant features (FDR-adjusted *p* < 0.05, ANOVA) based on >50 parts per million (ppm) abundance in ≥5% of samples. (E and F) Weight of 9 C57BL/6NTac lab mice, 14 C57BL/6NTac wildling, 15 former GF mice co-housed with lab mice (Lab Ex-GF), and 20 former GF mice co-housed with wildling mice (wildling Ex-GF) during (E) and after (F) the 10-week course of a choline-deficient 45% fat diet. The data are from 2–3 independent experiments. **p < 0.01 (Kruskal‒Wallis test with Benjamini‒Hochberg FDR correction). The means and SEM are shown.

We previously published that the wildlings' obesity-resistant phenotype requires colonization with wild-derived microbiota in the first two weeks of life,^18^ a time period during which both BAT^45^ and the immune system^46^ continue to develop. To determine whether the induction of the wildlings' phenotype depends on the age of the BAT or on the timing of the first interaction between the microbiota and the host's immune system, we transferred the microbiota of lab or wildling mice to adult germ-free (GF) mice via co-housing (Figure 5A). Adult GF mice have a fully developed BAT and a naïve immune system that has not yet been exposed to any microbiota. 16S rRNA gene profiling data from the microbiota in fecal pellets confirmed that adult GF mice successfully acquired lab or wildling microbiota. PCA at the level of the last known taxa (the highest resolution level) demonstrated tight and overlapping clusters of microbiota from wildling mice and co-housed Ex-GF mice, which were distinct from the equally tight and overlapping clusters of microbiota from lab mice and their co-housed Ex-GF mice (Figure 5B). The α-diversities of the microbiota from both wildling mice and their cohoused Ex-GF mice were significantly higher than those of the lab mice and their co-housed Ex-GF mice (Figure 5C). Unsupervised hierarchical analysis of the 16S data in a heatmap demonstrated that the microbiota of wildling mice and Ex-GF wildling mice were interspersed, whereas the microbiota of lab and Ex-GF lab mice formed a distinct group (Figure 5D).

The former GF mice (wildling Ex-GF and lab Ex-GF, respectively) were challenged with a 10-week course of high-fat diet. While wildling Ex-GF mice maintained a body weight similar to that of regular wildling mice on high-fat diet, lab Ex-GF mice gained significantly more body weight, similar to that of regular lab mice on high-fat diet (Figure 5E, F). These findings demonstrate that protection against diet-induced obesity is determined by the timing of the naïve immune system's initial encounter with the natural microbiota rather than by the developmental stage of brown adipose tissue.

### Protection against diet-induced obesity is not mediated by type 2 immune responses

**Figure 6.**
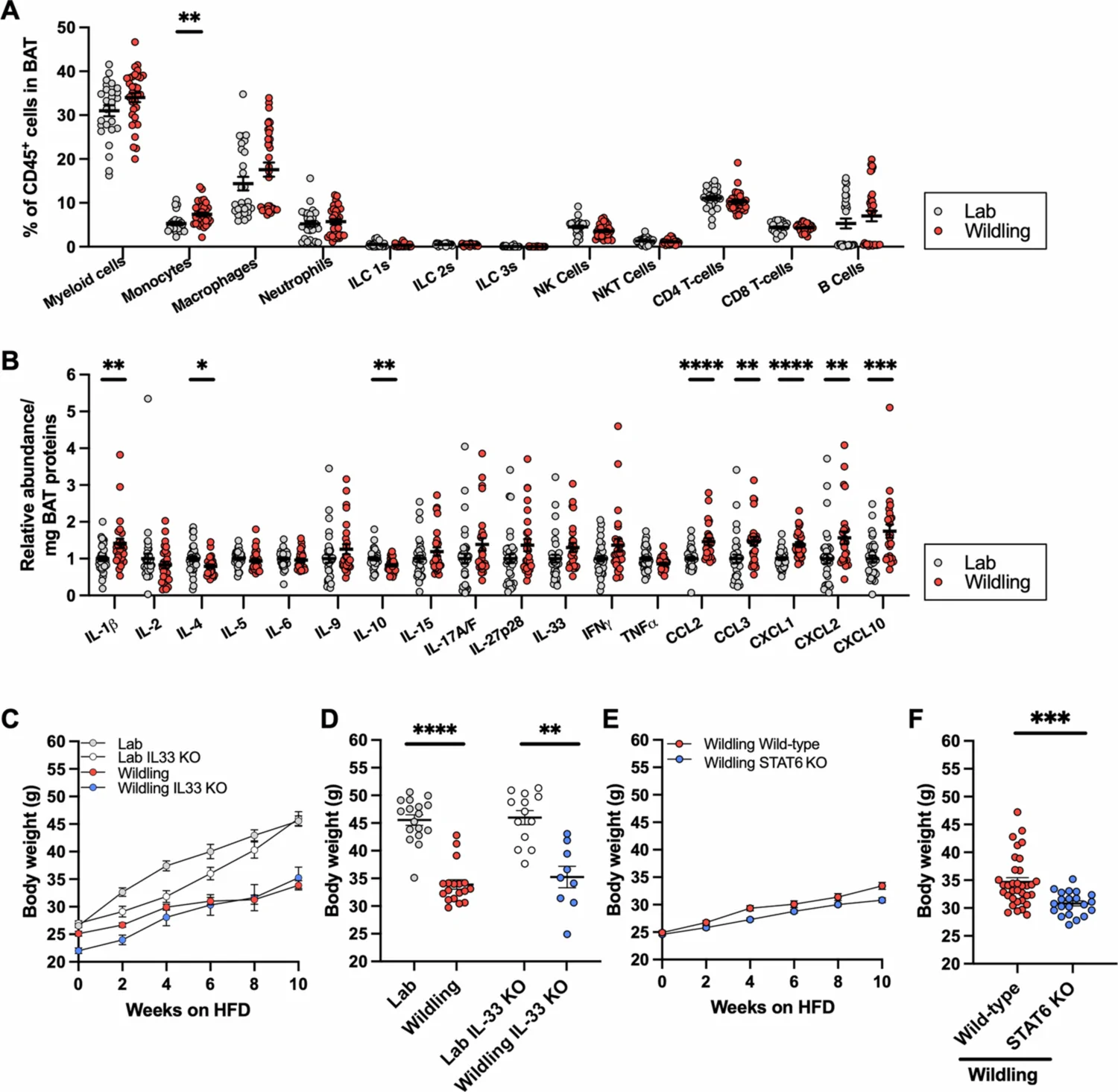
The wildlings' protection against diet-induced obesity is not mediated by type 2 immune responses. (A and B) Frequency of immune cell subsets within the CD45^+^ immune cell population (A) and relative cytokine/chemokine levels (B) in BAT of 12-day-old lab mouse pups and wildling mouse pups (*n* = 14–27 per group, data are from 1–3 independent experiments). (C–F) Weight of male lab (*n* = 17), wildling (*n* = 18), IL-33 KO lab (*n* = 13) and IL-33 KO wildling (*n* = 9) mice (C and D), and male wildling (*n* = 34) and STAT6 KO wildling (*n* = 21) mice (E and F) during (C and E) and after (D and F) a 10-week course of a choline-deficient 45% fat diet. ****p < 0.0001, ***p < 0.001, **p < 0.01, *p < 0.05. Mann‒Whitney tests with (A, B) or without (F) Benjamini‒Hochberg FDR correction) and the Kruskal‒Wallis test with Benjamini‒Hochberg FDR correction (D) were applied. The means and SEM are shown.

To identify immune events associated with the induction of the obesity-resistant phenotype, we characterized BAT immune cells and cytokines in lab and wildling pups at postnatal day 12. This time point is within the critical window of the immune system's initial exposure to the microbiota.^18^ At that time point, the BAT of wildling pups exhibited an increased frequency of monocytes compared to that of lab mouse pups, without any significant difference in the size of other innate and adaptive immune cell populations (Figure 6A, for FACS gating strategy, see Supplementary Figure S4). The BAT of wildling pups also showed significantly higher levels of IL-1β than BAT of lab mouse pups but significantly lower levels of the classical type 2 cytokines IL-4 and IL-10 (Figure 6B). Of note, all the tested chemokines, CCL2, CCL3, CXCL1, CXCL2, and CXCL10, were significantly increased in BAT of wildling pups compared to BAT of lab mouse pups (Figure 6B).

To determine the role of type 2 immune responses in the protection against diet-induced obesity, we generated IL-33 KO wildling mice and STAT6 KO wildling mice by co-housing the corresponding genetically modified lab mice with wildling mice. The generated L-33 KO and STAT6 KO wildling mice were bred in separate subcolonies, and the offspring were used for experiments to ensure that they had been exposed to the natural microbiota in early life.

As shown in Supplementary Figure S5A–C, the microbiota of IL-33 KO wildling mice clustered close to those of the wild-type wildling mice in a PCA analysis and separated from those of lab mice or IL-33 KO lab mice. Microbiota from IL-33 KO wildling mice also had a significantly higher α-diversity than those from IL-33 KO lab mice (Supplementary Figure S5B). Unsupervised hierarchical analysis of the 16S data in a heatmap demonstrated clear separation of microbiota from IL-33 KO lab mice and interspersion of microbiota from IL-33 KO wildling mice with those of the wildling mice (Supplementary Figure S5C). Further, IL-33 KO wildling mice tested positive for all commensals that were found in wild-type wildling mice except for *Spironucleus muris* (Supplementary Figure S5D).

Importantly, when challenged with a 10-week high-fat diet, IL-33 KO wildling mice displayed the same level of protection against diet-induced obesity as wildling mice that encoded IL-33 and gained significantly less weight than IL-33 KO lab mice (Figure 6C, D). Thus, the differential response of wildling mice and lab mice to a high-fat diet was not dependent on IL-33.

The microbiota of STAT6 KO wildling mice also clustered close to those of wild-type wildling mice and separated from those of SPF lab mice in a PCA analysis (Supplementary Figure S5A), but their α-diversity was lower than that of wildling mice (Supplementary Figure S5B), and there were bacterial taxa and commensals (*Helicobacter* *ganmani*, mouse norovirus, *Entamoeba muris*, and *T. muris*) that were not transferred (Supplementary Figure S5C, D). However, STAT6 KO wildling mice gained even less weight on a high-fat diet than wildling, which expressed STAT6 (*p* < 0.001, Figure 6E, F). Taken together, these results demonstrate that IL-33 and type-2 immune signaling are not required for the induction of the wildlings' protection against diet-induced obesity.

### CCR2 is required for microbiota-mediated protection against diet-induced obesity

Because of the increased size of the monocyte population (Figure 6A) and the abundance of the chemokine CCL2 (Figure 6B) in wildling BAT, we investigated whether CCL2-dependent recruitment of CCR2^+^ immune cells from the bone marrow was important for the wildlings' protection against diet-induced obesity. To answer this question, we created CCR2 KO wildling mice. The microbiota of CCR2 KO wildling mice clustered closely with those of wild-type wildling mice in a PCA analysis and exhibited the same level of α-diversity (Supplementary Figure S6). In contrast, the microbiota of CCR2 KO and wild-type lab mice clustered separately and exhibited significantly lower α-diversity (Supplementary Figure S6).

**Figure 7.**
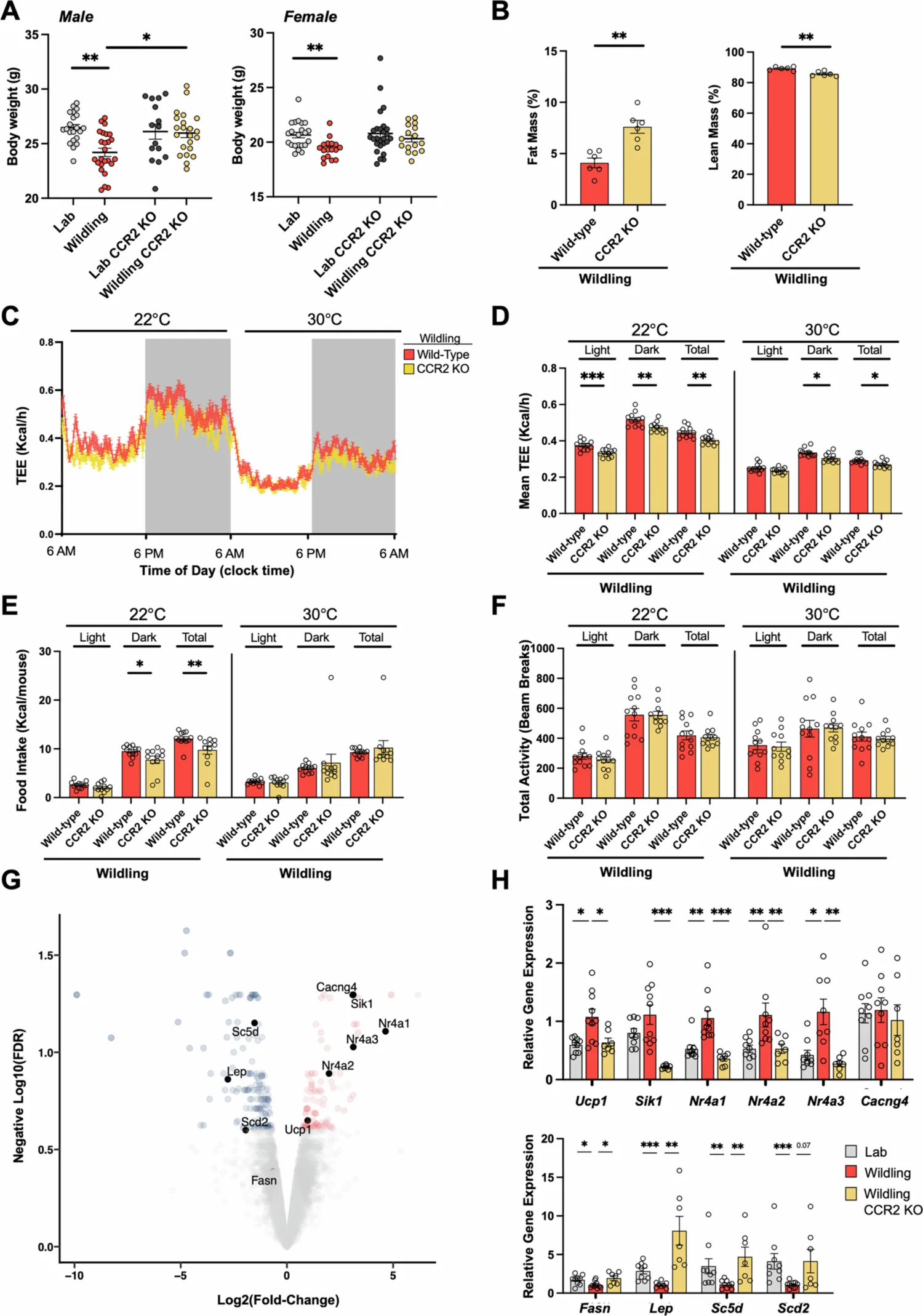
CCR2 is required for microbiota-mediated protection against diet-induced obesity. (A) Body weight of lab (21 male, 22 female), wildling (25 male, 17 female), lCCR2KO lab (15 male, 26 female), and CCR2 KO wildling ( 22 male, 16 female) mice at week 10–11 of life on an NIH-31 chow diet. Kruskall‒Wallis tests with Benjamini‒Hochberg FDR correction were applied. (B) Body composition (EchoMRI) of wild-type wildling and CCR2 KO wildling mice (6 males per group) at week 11–12 of life on an NIH-31 chow diet. (C–F) Male wild-type (*n* = 12) and CCR2 KO (*n* = 11) wildling mice were individually housed in metabolic cages at the indicated ambient temperature. The mice were 10–16 weeks of age on NIH-31 chow diet. The raw data were acquired over 48 hours. The total energy expenditure (C and D), food intake (E), and total activity (F) are shown per light or dark cycle. *p < 0.05, **p < 0.01, ***p < 0.01 (Mann‒Whitney test); (G) Volcano plot of differentially expressed genes from pseudobulk analysis of lab versus wildling brown adipocyte snRNA-seq data (cutoffs for significance are logCPM > 0.5, abs(logFC) > 0.5, FDR < 0.25). (H) Relative mRNA expression of selected genes in BAT from lab (*n* = 9), wildling (*n* = 8-10), and wildling CCR2 KO mice (*n* = 7). The data are from two independent experiments. *p < 0.05, **p < 0.01, ***p < 0.001. Mann‒Whitney tests were applied without (B, D, E, F) or with Benjamini‒Hochberg FDR correction (H). The means and SEM are shown.

While the absence of CCR2 had no significant effect on the weight of lab mice, male CCR2 KO wildling mice had significantly higher body weight, higher fat mass, and lower lean mass than wild-type wildling mice on NIH chow diet (Figure 7A, B).

To examine the metabolic phenotype, we measured energy expenditure via indirect calorimetry. Consistent with the results of weight gain, male CCR2 KO wildling mice displayed significantly lower energy expenditure than wild-type wildling mice (Figure 7C, D). They consumed less food but had the same physical activity as the wild-type wildling mice (Figure 7E, F). When housed at 30 °C, energy expenditure decreased significantly for all the mice but was still lower in CCR2 KO wildling mice than in the wild-type wildling mice (Figure 7C, D), with no difference in food consumption or physical activity (Figure 7E, F). These results demonstrate that the natural microbiota cannot induce the wildling metabolic phenotype in mice that do not express CCR2.

To assess the transcriptional profile of adipocytes from BAT of CCR2 KO wildling mice compared to those of wildling and lab mice, we isolated nuclei from BAT of CCR2 KO wildling mice and evaluated by qPCR the mRNA levels of selected genes that were differentially expressed in lab and wildling adipocytes (Figure 7G). Just like lab mice, CCR2 KO wildling mice had significantly lower expression of *Ucp1*, *Nr4a1*, *Sik1*, *Nr4a2*, and *Nr4a3* transcripts in BAT than wild-type wildling mice (Figure 7H). These findings are consistent with the snRNAseq results of the mAd4 population, which is dominant in wildling mice (Figure 4F, G), and with the results of the pseudobulk analysis (Figure 7G). Likewise, similar to lab mice, CCR2 KO wildling mice had significantly higher expression of *Fasn*, *Lep*, and *Sc5d* and a trend of higher expression of *Scd2* in BAT than wild-type wildling mice. The differential expression of *Fasn* was consistent with the snRNA-seq results of the mAd1 population, which is dominant in lab mice compared to wildling mice (Figure 4E, G). Thus, fat and lean body mass, increased energy expenditure, and the unique transcriptome of BAT adipocytes from wildling mice all depend on CCR2 expression.

### Protection against diet-induced obesity depends on CCR2-mediated recruitment of monocytes

**Figure 8.**
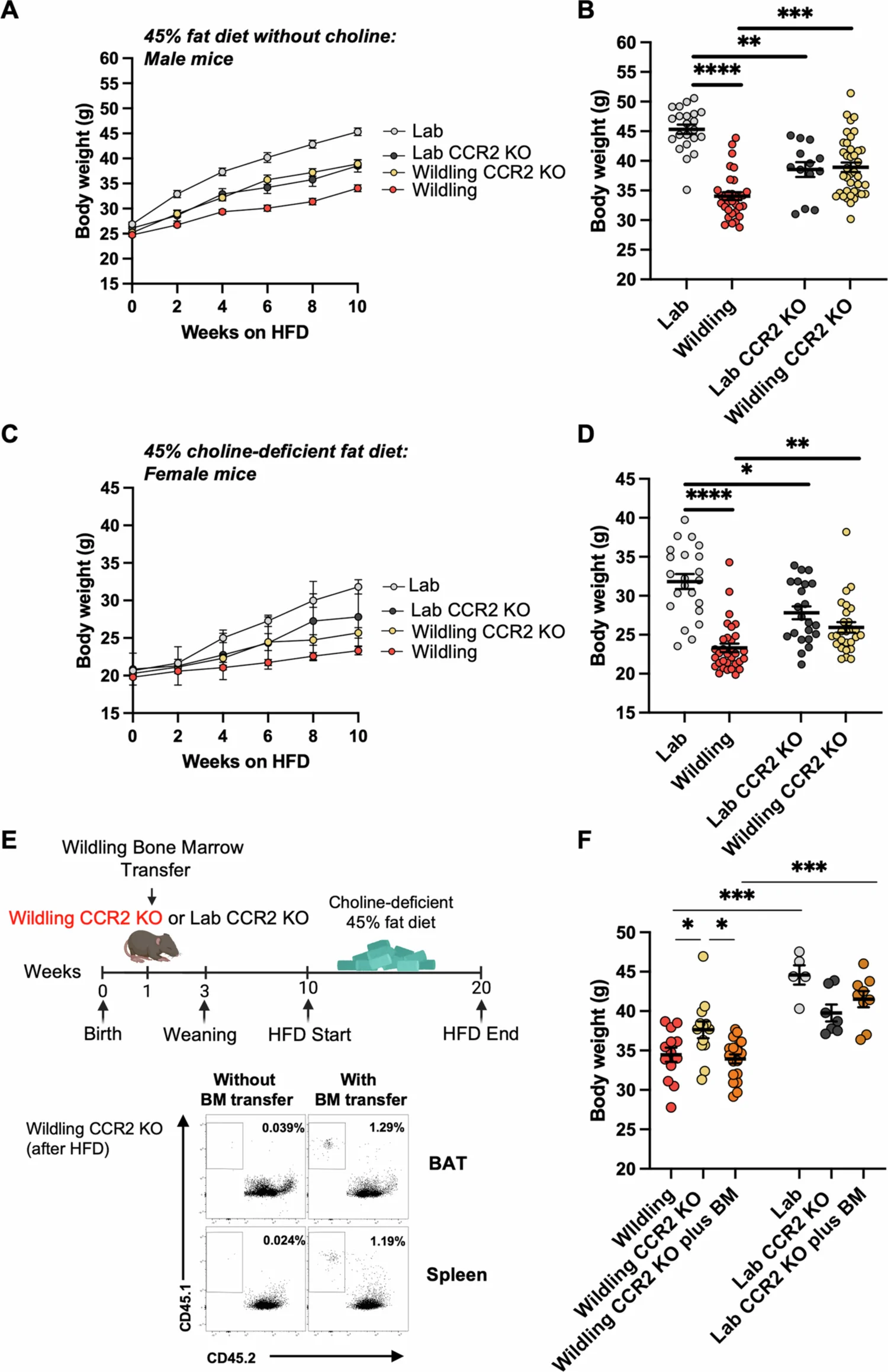
Early-life transfer of bone marrow into CCR2 KO wildling mice restores protection against diet-induced obesity. (A–D) Body weight of male (A, B) and female (C, D) lab (22 male, 22 female), wildling (32 male, 32 female), CCR2 KO lab (13 male, 22 female) and CCR2 KO wildling (39 male, 27 female) mice during (A and C) and after (B and D) a 10-week course of a choline-deficient 45% fat diet. (E) Experimental design of the bone marrow transfer studies. Seven- to ten-day-old CD45.2^+^ pups of CCR2 KO wildling mice or CCR2 KO lab mice did or did not receive an i.p. injection of wildling bone marrow and were challenged with a choline-deficient 45% fat diet. The representative flow cytometry plots indicate the presence of the transferred CD45.1^+^ cells in the spleen and BAT of CD45.2^+^ CCR2 KO lab mice after week 20 of life, which was after a 10-week course of a choline-deficient 45% fat diet. (F) Body weight of the indicated mice after choline-deficient 45% fat diet. Male CCR2 KO wildling mice had (*n* = 19) or had not (*n* = 13) received bone marrow from wildling mice at day 10–12 of life. Male CCR2 KO lab mice had (*n* = 9) or had not (*n* = 7) received bone marrow from wildling mice at days 10–12 of life. Wildling mice (*n = 13*) and lab mice (*n = 5*) served as controls. ****p < 0.0001, ***p < 0.001, **p < 0.01, *p < 0.05. Kruskall‒Wallis tests with Benjamini‒Hochberg FDR correction (B, D) or Brown‒Forsythe and Welch tests with Benjamini‒Hochberg FDR correction (F) were applied. HFD, high-fat diet; CCR2, C-C motif chemokine receptor 2; BM, bone marrow. BioAnalytics) using pooled samples from 5–6 mice per group.

When challenged with a 10-week course of high-fat diet, male CCR2 KO wildling mice were not protected from obesity and exhibited significantly higher body weight than male wild-type wildling mice, with a similar trend observed in female CCR2 KO wildling mice. The opposite was observed in lab mice, where a lack of CCR2 expression resulted in a significant reduction in weight gain (Figure 8A–D). Thus, in the absence of CCR2, lab and wildling mice experienced the same weight gain.

To examine whether the obesity-resistant phenotype can be restored in CCR2 KO wildling mice, we reconstituted them with bone marrow from wild-type wildling mice. To avoid irradiating the recipient mice, which would have altered the microbiota,^47^ we transferred bone marrow early in life to 7–10-day-old CCR2 KO wildling mouse pups. The pups received wildling bone marrow via intraperitoneal injection and were challenged at week 10 of life with a 10-week course of high-fat diet (Figure 8E). At the conclusion of the high-fat diet course, CD45.1^+^ cells from the transferred bone marrow were detected via flow cytometry in both the spleen and BAT of the recipient CD45.2^+^ CCR2 KO mice at week 20 of life (Figure 8E).

Bone marrow transfer significantly reduced the weight gain of CCR2 KO wildling mice compared to CCR2 KO wildling mice that had not received bone marrow (Figure 8F, orange vs. yellow symbols). In fact, at the end of the high-fat diet challenge, the body weight of the bone marrow-reconstituted CCR2 KO wildling mice was as low as those of the wild-type wildling mice (Figure 8F, orange vs. red symbols). In contrast, CCR2 KO lab mice that had received the same bone marrow gained significantly more weight than CCR2 KO wildling mice (Figure 8F, brown vs orange).

Collectively, these results demonstrate that CCR2^+^ cells are required for protection against diet-induced obesity.

## Discussion

There is an emerging consensus^12^ ^,^ ^15^ that the addition of natural microbiota, commensals and pathogens from wild mice to conventional lab mice improves the study of immune responses in preclinical disease models.^16^ ^,^ ^48-50^ To facilitate this research, we have previously generated a colony of C57BL/6 mice with natural microbiota and pathogens, the founders of which were C57BL/6 mice born to wild mice.^17^ We have characterized the naturalized immune responses in this model and demonstrated that they remain stable over years in a standard animal facility.^16^ ^,^ ^17^ We now demonstrate that the same is true for the metabolic phenotype.

While it is well established that early-life disruption of the microbiota predisposes to obesity later in life,^4^ we show here that early-life colonization with natural microbiota and commensals confers life-long protection against diet-induced obesity. This protection is observed across a spectrum of diets, diverse genetic backgrounds, and it is independent of sex. Several lines of evidence link the protective metabolic phenotype to BAT activation. Using indirect calorimetry, we demonstrated increased energy expenditure when wildling mice were held at room temperature and a significant decrease in energy expenditure at thermoneutrality. This was associated with the differential transcriptomic profile of brown adipocytes, which were separated into distinct clusters in wildling and lab mice. Brown adipocytes of wildling mice had upregulated expression of genes such as *Ucp1*, *Gnas*, *Ppara, Acsl1,* and *Cpt1b* transcripts​​​​, which encode proteins relevant for thermogenesis and fatty acid *β*-oxidation,^36^ ^,^ ^51-54^ whereas those of lab mice were enriched in pathways that are associated with increased fatty acid biosynthesis and an increased lipogenesis.^42^ This unique transcriptional profile of BAT of wildling mice was not observed in CCR2 KO wildling mice.

We confirm a previous study that adipocytes in BAT can be heterogeneous with respect to their thermogenic activity,^55^ and identified a major population of thermogenic adipocytes that are induced in response to colonization with wild-derived microbiota. Consistent with this notion, adipocytes from lab mice and wildling mice were clearly separated in an integrated UMAP. These findings are of translational relevance because increased BAT activity is also associated with improved metabolic parameters in humans,^19^ opening the possibility that this can be achieved by modification of the microbiota. Together, these data and the stability of the metabolic phenotype render the wildling colony an interesting and useful model for metabolic studies.

We previously reported that lab mice acquire an obesity-resistant phenotype only if they are exposed to the wildling microbiota during the first two weeks of life.^18^ We now show that an active immune response occurs in this window of time, with increased levels of chemokines and monocytes in BAT of wildling mice compared to BAT of lab mice. These results are reminiscent of the weaning reaction, which links early-life exposure to lab mouse microbiota with immune system maturation in the gut, lung and skin.^56^ ^,^ ^57^ The obesity-resistant phenotype can also be induced in adult GF mice with a naïve and immature immune system via exposure to the wild-derived microbiota. In contrast, lab mouse microbiota do not induce an obesity-resistant phenotype. Thus, we demonstrate the first interaction between the microbiota and the immune system rather than the developmental age of BAT as a critical factor for the establishment of a beneficial metabolic phenotype.

Type-2 immune responses are well known as regulators of BAT and WAT function and homeostasis.^58-60^ For example, a series of studies demonstrated that type-2 immune responses promote beiging of WAT and the thermogenic activity of BAT, in some cases in an IL-33-dependent manner.^61-63^ These effects have been linked to the microbiota^64^ and to symbionts such as helminths.^64-66^ Indeed, type-2 immune response-inducing symbionts such as Kazachstania pintolopesii^67^ and skin mites are present in wildling mice.^17^ However, type-2 immune responses do not contribute to the metabolic phenotype described here because resistance against diet-induced obesity is preserved in STAT6 KO wildling mice. Rather, we attribute the beneficial metabolic phenotype to an increased myeloid tone in BAT.

The loss of the metabolic phenotype and the weight gain of CCR2 KO wildling mice point to a role of myeloid cells that are recruited from the bone marrow. CCR2, while expressed on many leukocytes, is most highly expressed on monocytes.^68^ Bone marrow-derived monocytes replace fetal liver-derived macrophages in BAT and other tissues in early postnatal life and develop into tissue-resident macrophages,^69^ suggesting a causal role of CCR2^+^ monocytes in the metabolic phenotype of wildling mice. Indeed, an increased concentration of CCL2 and an increased size of the monocyte population are found in 12-day-old wildling pups as compared to lab mouse pups. Bone marrow-derived monocytes continue to replace BAT macrophages during adult life,^69^ which may be the reason that the metabolic phenotype of wildlings can be induced in adult GF mice via microbiota transfer. Because CCR2 also affects many processes outside BAT, further studies are warranted to identify the precise mechanism that induces the observed phenotype of wildling mice.

In conclusion, our study provides strong evidence of the existence of a natural microbiota–immune system–adipose tissue axis, which is established in early life and exerts life-long beneficial effects on host metabolism.

## Supplementary Material

Supplementary Material Suppl_Fig_6.jpg

Supplementary Material Suppl_Fig_5.jpg

Supplementary Material Suppl_Fig_4.jpg

Supplementary Material Suppl_Fig_3.jpg

Supplementary Material Suppl Figure 2.png

Supplementary Material Suppl Figure 1.png

Supplementary Material Supplementary Table 1.docx

Supplementary Material Supplementary_Figure_clean.docx

## Data Availability

Raw sequence data from 16S rRNA gene profiling are deposited in the NCBI Sequence Read Archive under BioProject number PRJNA1492208, and the snRNAseq results in NCBI Gene Expression Omnibus (GEO) and the Broad Institute Single Cell Portal (https://singlecell.broadinstitute.org/single_cell).
