# Supplementary material for: Natural microbiota confer life-long protection against obesity via an early-life, immune-mediated effect on brown adipocytes
Source: Gut Microbes. 2026 Aug 23;18(1):2718613. doi: 10.1080/19490976.2026.2718613 (PMC13505454; doi:10.1080/19490976.2026.2718613)
Supplement: Supplementary file 1 [file KGMI_A_2718613_SM8237.docx]

**Legends to Supplementary Figures**

**Supplementary Figure S1. 16*S* rRNA gene profiling of microbiota in fecal pellets from wildling and lab mice on various genetic backgrounds.**

The heat map, generated by unsupervised hierarchical clustering, shows the top 50 most variant features (FDR-adjusted *P* < 0.05, ANOVA) based on >50 parts per million (ppm) abundance in ≥5% of samples. The heat-map displays last-known taxa (LKT), i.e. the lowest, most specific ranks that can be unambiguously identified. Wildling mice on the B6.SJL and Balb/c background were created as independent colonies whose founders were B6.SJL and Balb/c lab mice co-housed for four weeks with C57BL/6NTac wildling mice.

**Supplementary Figure S2. Single nuclei RNA sequencing analysis of brown adipocytes from lab and wildling mice housed at high ambient temperature.**

**A,** Uniform manifold approximation and projection (UMAP) plot of an integrated analysis of single-nuclei RNA sequencing data of interscapular BAT from two lab and one wildling mice housed at high (26-28°C, thermoneutrality) ambient temperature and the relative size of the identified populations.  Nuclei are colored by cell type; grey indicates nuclei from other conditions. Bar graphs show the relative size of the identified population. Lab, 14,632 nuclei; wildling, 7,316 nuclei. ASPC, adipocyte stem and progenitor cells; LEC, lymphatic endothelial cells; SMC, smooth muscle cells.

**B**, Violin plots showing expression of selected marker genes.

**C**, UMAP plot of adipocyte nuclei from BAT of lab and wildling mice housed at high temperature (26°-26°C). Bar graphs show the relative size of the identified populations. Nuclei in the plotted condition are colored by cell type; light grey cells in the left UMAP represent cells from wildling mice, and light grey cells in the right UMAP represent cells from lab mice. Lab,13,342 nuclei; l, 6395 nuclei.

**D**, Violin plots of marker genes for each subcluster.

**Supplementary Figure S3. Natural microbiota reduce the expression of thermogenic genes in inguinal white adipose tissue.**

Inguinal white adipose tissue (iWAT) from 10–16-week-old mice, fed with NIH-31 chow diet, was analyzed.

**A,** Weight of iWAT from 23 lab and 26 wildling mice (data from three independent experiments).

**B**, Relative expression of *Lep* in iWAT from lab and wildling mice (data from six independent experiments).

**C**, Leptin levels in sera of 20 lab and 23 wildling mice (data from two independent experiments).

**D**, Hematoxylin and eosin (H&E)-stained iWAT (representative of iWAT from 12 wildling and 12 lab mice studied in two independent experiments) and quantification of lipid droplets (5 iWAT sections per mouse from 7 wildling and 7 lab mice). Original magnification x 20; scale bars, 50 µm.

**E,** Relative mRNA expression of selected thermogenic genes in iWAT from 18-42 lab and 20-45 wildling mice (data from three to six independent experiments).

**F**, Relative mRNA expression of creatinine futile cycle genes in iWAT from 38-39 lab and 42 wildling mice (data from five independent experiments).

**** *P* < 0.0001, *** *P* < 0.001, ** *P* < 0.01, * *P* < 0.05 (Mann-Whitney tests without (A-D) or with (E,F) Benjamini-Hochberg FDR correction).

**Supplementary Figure S4. Flow cytometry of BAT immune cell populations.**

Flow cytometry gating strategy for profiling of immune subsets in BAT of lab and wildling mouse pups.

**Supplementary Figure S5. Gut microbial composition of wildling mice, IL-33 KO wildling mice, STAT6 KO wildling mice, lab mice and IL-33 KO lab mice.**

16S rRNA gene profiling was performed on fecal pellets from 10 lab mice, five IL-33 KO lab mice, 10 wildling mice, five IL-33 KO wildling mice and five STAT6 KO wildling mice (all mice were C57BL/6NTac). IL-33 KO and STAT6 KO wildling mice were created and maintained as independent colonies whose founders were IL-33 KO and STAT6 KO lab mice, co-housed for four weeks with C57BL/6NTac wildling mice.

**A,** PCA of last known taxa (LKT), normalized to number of bases for analysis in sample and log2 transformed, with the Bray dissimilarity index applied. LKT are the lowest, most specific ranks that can be unambiguously identified. Ellipses indicate 90% confidence regions. *P* < 0.0001 (PERMANOVA); F−Model = 14.2312.

**B,** Chao 1 ⍺-diversity index. *** *P* < 0.001, ** *P* < 0.01, * *P* < 0.05 (Kruskall-Wallis test with Benjamini-Hochberg FDR correction).

**C,** LKT level heat map, generated by unsupervised hierarchical clustering, shows the top 50 most variant features (FDR-adjusted *P* < 0.05, ANOVA) based on >50 parts per million (ppm) abundance in ≥5% of samples. LKT-level heat map, generated by unsupervised hierarchical clustering, showing the top 50 most variant features (FDR-adjusted *P* < 0.05, ANOVA) based on >50 parts per million (ppm) abundance in ≥5% of samples.

**D,** Results of pathogen screening by real-time PCR on fecal pellets and skin swabs (IDEXX BioAnalytics) using pooled samples from 5 mice per group.

**Supplementary Figure S6. Gut microbial composition and pathogen profile of wildling mice, CCR2 KO wildling mice, lab mice and CCR2 KO lab mice.**

16S rRNA gene profiling was performed on fecal pellets from 10 C57BL/6NTac lab mice (lab wild-type), 11 CCR2 KO lab mice, 10 C57BL/6NTac wildling mice (wildling Wild-type) and 12 CCR2 KO wildling mice. The wildling mice on the CCR2 KO background were created and maintained as a separate colony whose founders were CCR2 KO lab mice co-housed for four weeks with C57BL/6NTac wildling mice.

**A,** PCA of last known taxa (LKT), normalized to number of bases for analysis in sample and log2 transformed, with the Bray dissimilarity index applied. LKT are the lowest, most specific ranks that can be unambiguously identified. Ellipses indicate 90% confidence regions. Ellipses indicate 90% confidence regions *P* < 0.0001 (PERMANOVA); F−Model =17.0332.

**B,** Chao 1 ⍺-diversity index. ** *P* < 0.01 *, *P* < 0.05 (Kruskall-Wallis test with Benjamini-Hochberg FDR correction).

**C,** LKT-level heat map, generated by unsupervised hierarchical clustering, showing the top 50 most variant features (FDR-adjusted *P* < 0.05, ANOVA) based on >50 parts per million (ppm) abundance in ≥5% of samples.

**D,** Results of pathogen screening by real-time PCR on fecal pellets and skin swabs (IDEXX
