# Supplementary material for: Natural microbiota confer life-long protection against obesity via an early-life, immune-mediated effect on brown adipocytes
Source: Gut Microbes. 2026 Aug 23;18(1):2718613. doi: 10.1080/19490976.2026.2718613 (PMC13505454; doi:10.1080/19490976.2026.2718613)
Supplement: Supplementary file 2 [file KGMI_A_2718613_SM8239.docx]

Table S1. Primer list

| Gene | Species | Froward primer 5’ 🡪 3’ | Reverse primer 5’ 🡪 3’ |
| --- | --- | --- | --- |
| β-Actin | Mouse | GCCTCACTGTCCACCTTCCA | GGGCCGGACTCATCGTACT |
| β-Actin | Mouse | CCTTCTTGGGTATGGAATCCTGT | CACTGTGTTGGCATAGAGGTCTTTAC |
| Rpl7 | Mouse | ACGGTGGAGCCTTATGTGAC | TCCGTCAGAGGGACTGTCTT |
| 18S | Mouse | GGCCGTTCTTAGTTGGTGGAGCG | CTGAACGCCACTTGTCCCTC |
| UCP-1 | Mouse | ACTGCCACACCTCCAGTCATT | CTTTGCCTCACTCAGGATTGG |
| ADRB3 | Mouse | TCTTGGGGCAACCAGTCAAG | CACCGCTCAACAGGTTTGA TG |
| Cidea | Mouse | TGACATTCATGGGATTGCAGAC | GGCCAGTTGTGATGACTAAGAC |
| Cidec | Mouse | ATGGACTACGCCATGAAGTCT | CGGTGCTAACACGACAGGG |
| DIO2 | Mouse | CAGTGTGGTGCACGTCTCCAA TC | TGAACCAAAGTTGACCACCAG |
| Leptin | Mouse | GAGACCCCTGTGTCGGTTC | CTGCGTGTGTGAAATGTCATTG |
| PGC1a | Mouse | AGCCGTGACCACTGACAACGAG | GCTGCATGGTTCTGAGTGCTAAG |
| PRDM16 | Mouse | GCCATGTGTCAGATCAACGA | CCTTCTTTCACA TGCACCAA |
| Alpl | Mouse | TCAACACCAATGTAGCCAAGA | GTAGCTGGCCCTTAAGGATTC |
| CKB | Mouse | GCCTCACTCAGATCGAAACTC | GGCATGTGAGGATGTAGCCC |
| Gatm | Mouse | GCTTCCTCCCGAAATTCCTGT | CCTCTAAAGGGTCCCATTCGT |
